# Uterine fibroids are associated with increased risk of pre-eclampsia: A case-control study

**DOI:** 10.3389/fcvm.2022.1011311

**Published:** 2022-10-18

**Authors:** Lina Gong, Meng Liu, Haiheng Shi, Ying Huang

**Affiliations:** Obstetrics Department, People's Hospital of Xinjiang Uygur Autonomous Region, Urumqi, China

**Keywords:** pre-eclampsia, uterine fibroids, pelvic tumor, pregnant women, risk

## Abstract

**Purpose:**

Uterine fibroids are associated with hypertension in non-pregnant women. We aimed to evaluate the association between uterine fibroids and pre-eclampsia (PE).

**Patients and methods:**

Participants were pregnant women who delivered in the Department of Obstetrics of the People's Hospital of Xinjiang Uygur Autonomous Region between January and December 2021. Patients with PE were identified as the case group, whereas those without PE were selected as the control group, using age-matching and a ratio of 1:5. Ultrasound examination during early pregnancy was used to detect uterine fibroids. Multivariable logistic regression was applied to evaluate the association between uterine fibroids and PE.

**Results:**

In total, 121 cases with PE and 578 controls without PE were included, with mean age of 32.9 years and gestational age of 37.7 weeks. Time of ultrasound examination was 12.0 ± 2.6 weeks. The case group had a significantly higher exposure rate of uterine fibroids than the control group (14.0 vs. 6.9%, *P* = 0.009). Multivariable Logistic regression models adjusted for potential confounding factors, including gestational age and blood pressure in early gestation, showed that pregnant women with uterine fibroids in early pregnancy exhibited three-fold higher odds for PE (OR, 3.02; 95% CI, 1.20–7.60; *P* = 0.019). Sensitivity analysis, which excluded those with gestational diabetes, further confirmed the robustness of the results. The association between uterine fibroids and PE was stronger in pregnant women aged ≥35 years and multiparas.

**Conclusion:**

Uterine fibroids are significantly associated with an increased risk of PE in pregnant women. Uterine fibroids may serve as a new factor for identifying pregnant women at high risk of PE, and the effect of myomectomy before pregnancy on prevention of PE is worth further exploring.

## Introduction

Pre-eclampsia (PE) affects 3–5% of pregnant women and remains a major cause of maternal and perinatal morbidity and mortality worldwide (1, 2). Women with PE have a more than two-fold increased risk of obstetric complications (3). PE is associated with severe maternal events, such as cardio cerebrovascular disease, pulmonary edema, and liver and kidney failure (4). Moreover, it is related to abortion, preterm birth, fetal growth restriction, and stillbirth (5). In addition, the risk of long-term postpartum adverse events is increased in women with a history of PE and in their offspring (6, 7). Termination of pregnancy is currently the main treatment for PE, although it has several disadvantages. Early identification of high-risk pregnant women has maternal and fetal benefits. However, the predictive power of traditional risk factors for PE is unsatisfactory, suggesting that more risk factors should be explored (8).

Uterine fibroids are the most common type of benign pelvic tumor in women, with a prevalence of 20–40% (9, 10). Several studies have demonstrated that the risk of hypertension is higher in women with uterine fibroids than in those without (11–13). A recent meta-analysis further confirmed these results (14). The authors pooled 10 studies with 8,361 participants and showed that women with fibroids had a 44% increased risk of hypertension. Uterine fibroids are generally asymptomatic, and most patients are not detected until early pregnancy by obstetrical ultrasound. The prevalence of uterine fibroids complicated with pregnancy ranges from 3 to 12% (15). Given the partial overlap of pathogeneses of hypertension and PE, such as oxidative stress and endothelial dysfunction, we hypothesized that the presence of fibroids is associated with the occurrence and development of PE. In addition, the effects of fibroids on blood pressure may be amplified by the rapid expansion of fibroids during pregnancy (16, 17). However, the association between uterine fibroids and PE has rarely been reported. Therefore, we conducted this case-control study to evaluate this association.

## Materials and methods

### Study subjects

Participants of this study were pregnant women admitted to the People's Hospital of the Xinjiang Uygur Autonomous Region for delivery between January and December 2021. We selected patients who were diagnosed with PE as cases. The inclusion criteria were aged at least 18 years old, singleton delivery with a live newborn, and available ultrasound results of early pregnancy. The exclusion criteria were a history of hypertension or diabetes before gestation, diagnosis of malignant tumor, or *in vitro* fertilization. For the evaluation of the association between uterine fibroids and PE, we further excluded those with blood pressure (BP) ≥140/90 mmHg before 20 weeks' gestation. Pregnant women with normal BP level and without other signs of PE were identified using a case-control ratio of 1:5, matched for maternal age and set as the control group. This study was conducted in accordance with the Declaration of Helsinki and approved by the Ethics Committee of the People's Hospital of Xinjiang Uygur Autonomous Region. All participants or legal representatives signed a written consent form.

### Data collection and definition of disease

Data were retrieved and collected using a predesigned spreadsheet from the electronic medical record system, including demographic information (maternal age and ethnicity), physical examination (height, weight, and blood pressure), laboratory tests [fasting plasma glucose (FPG), total cholesterol, triglyceride, high-density lipoprotein cholesterol, and low-density lipoprotein cholesterol], and obstetric history. Seated BP was measured twice in the upper arm by trained nurses after patients rested quietly for at least 10 min, according to the standard measurement procedure. The mean values of two measurements were used for the analysis. BP in early gestation (< 20 weeks of gestation) were collected from outpatient records or self-reported by the pregnant women. Body mass index (BMI) was calculated as weight (kg) divided by height (m) squared. Dyslipidemia was defined as abnormal lipid levels at baseline due to pregnancy.

The exposure factor was uterine fibroids, which was defined as the presence of at least one fibroid on early pregnancy ultrasound examination (*n* = 45) or a self-reported history of fibroids (diagnosed by ultrasound before pregnancy, *n* = 12) that had not undergone myomectomy. PE was defined according to the ISSHP recommendation as systolic BP (SBP) ≥140 mmHg and/or diastolic BP (DBP) ≥90 mmHg after 20 weeks gestation, combined with at least one of the following new-onset conditions: (1) positive proteinuria (protein–to–creatinine ratio of ≥30 mg/mmol or urinary dipstick ≥2+); (2) other maternal organ dysfunction (serum creatinine ≥90 μmol/L, alanine aminotransferase or aspartate aminotransferase >40 IU/L); (3) neurological complications (eclampsia, altered mental state, blindness, stroke, clonus, severe headaches, or persistent visual scotomata); (4) hematological complications (thrombocytopenia-platele count < 150,000/μL, disseminated intravascular coagulation, and hemolysis); and (5) uteroplacental dysfunction (fetal growth restriction, abnormal umbilical artery Doppler wave form analysis, or stillbirth) (18).

### Statistical analysis

Continuous variables are presented as mean ± standard deviation or median (interquartile range) and were compared between groups using Student's *t*–test or Mann–Whitney U test according to the results of the normality test. Categorical variables were summarized as numbers and percentages and compared between the groups using Pearson's Chi-square test. Multivariable logistic regression models were used to evaluate the association between uterine fibroids and PE. Odds ratios (OR) with 95% confidence intervals (CI) for outcomes were estimated for pregnant women with uterine fibroids by comparing them with those without uterine fibroids. To evaluate the independent association, demographic variables, including age, BMI, and ethnicity, were first adjusted in the logistic regression model. FPG, dyslipidemia, and serum creatinine levels were further adjusted as these factors have been associated with BP changes. In addition, age at menarche and history of gestation were included in the adjustment model. Finally, gestational age and BP in early gestation were further considered. Tolerance and variance inflation factors were used for collinearity testing among the included variables.

To evaluate the robustness of the results, we conducted sensitivity analyses by excluding pregnant women with gestational diabetes, dyslipidemia and those with self-reported fibroids. Furthermore, interaction terms were introduced into the multivariable model to evaluate whether the association between uterine fibroids and PE differed according to maternal age (< 35 or ≥35 years old), BMI (< 28 or ≥28), primipara (yes or no), and dyslipidemia (yes or no). Statistical analyses were performed using IBM SPSS Statistics for Windows version 23.0 (IBM Corp., Armonk, N.Y., USA). Two-tailed *P* < 0.05 were considered statistically significant.

## Results

### Characteristics of the participants

A total of 121 pregnant women diagnosed with PE were identified as the case group. Accordingly, 578 age-matched pregnant women who had normal BP levels and no other signs of PE were selected as controls. Therefore, a total of 699 participants were included in this study, with mean age and BMI of 32.9 ± 4.2 years and 28.2 ± 4.2 kg/m^2^, respectively. Moreover, 415 (59.4%) were primipara, and the mean time of ultrasound examination was 12.0 ± 2.6 weeks. Table 1 shows that the case group had significantly higher BMI and BP levels and a higher proportion of dyslipidemia. Exposure rate to uterine fibroids in the case group was twice that of the control group (14.0 vs. 6.9%, *P* = 0.009).

**Table 1:** Characteristics of study participants.

**Characteristics**	**Overall**	**Case**	**Control**	***P-*value**
	**(*n* = 699)**	**(*n* = 121)**	**(*n* = 578)**	
Age (year)	32.9 ± 4.2	33.0 ± 4.4	32.9 ± 4.2	0.797
BMI (kg/m^2^)	28.2 ± 4.2	30.8 ± 4.5	27.6 ± 4.0	< 0.001
SBP (mmHg)	119.3 ± 17.8	145.3 ± 21.3	113.9 ± 10.8	< 0.001
DBP (mmHg)	76.6 ± 12.6	94.6 ± 13.8	72.8 ± 8.3	< 0.001
SBP in early gestation	109.8 ± 11.6	121.0 ± 14.1	107.5 ± 9.5	< 0.001
DBP in early gestation	70.5 ± 9.2	79.9 ± 9.9	68.5 ± 7.7	< 0.001
Ethnicity, n (%)				
Han	339 (48.5)	43 (35.5)	296 (51.2)	0.002
Others	360 (51.5)	78 (64.5)	282 (48.8)	
FPG (mmol/L)	4.9 ± 1.1	4.9 ± 1.1	4.9 ± 1.1	0.839
Total cholesterol (mmol/L)	5.8 ± 1.4	6.2 ± 1.6	5.7 ± 1.2	0.004
Triglyceride (mmol/L)	3.6 ± 1.6	3.9 ± 1.6	3.5 ± 1.6	0.013
HDL-C (mmol/L)	1.7 ± 0.6	1.7 ± 0.6	1.7 ± 0.6	0.296
LDL-C (mmol/L)	2.8 ± 1.2	3.3 ± 1.4	2.7 ± 1.1	< 0.001
Dyslipidemia, *n* (%)	304 (43.5)	97 (80.2)	207 (35.8)	< 0.001
Serum creatinine (μmol/L)	46.9 ± 9.8	55.2 ± 13.3	45.3 ± 7.9	< 0.001
Age at menarche (year)	12.5 ± 1.0	12.5 ± 0.9	12.5 ± 1.0	0.745
Primipara, *n* (%)	415 (59.4)	79 (65.3)	336 (58.1)	0.145
Uterine fibroids, *n* (%)	57 (8.2)	17 (14.0)	40 (6.9)	0.009
Time of ultrasound examination (weeks)	12.0 ± 2.6	13.9 ± 3.1	11.6 ± 2.3	< 0.001
Gestational age (weeks)	37.7 ± 2.8	35.9 ± 3.6	38.0 ± 2.4	< 0.001

Data are presented as means ± standard deviation or median (interquartile range) or number (percentage). BMI, body mass index; SBP, systolic blood pressure; DBP, diastolic blood pressure; FPG, fasting plasma glucose; HDL-C, high-density lipoprotein cholesterol; LDL-C, low-density lipoprotein cholesterol.

### Association between uterine fibroids and PE

Unadjusted logistic analysis revealed that pregnant women with uterine fibroids had 1.2-fold increased odds for the presence of PE (OR, 2.20; 95% CI, 1.20–4.03; *P* = 0.011). Results remained unchanged after adjusting for maternal age, BMI, ethnicity, FPG, dyslipidemia, serum creatinine, age at menarche, and primipara (Table 2, models 2 and 3). When gestational age at delivery and BP in early gestation was further adjusted, the association of uterine fibroids with PE was stronger. In the full-adjusted model, women with uterine fibroids showed three-fold increased odds for PE than those without uterine fibroids (model 5). No obvious collinearity was detected among the variables in the fully adjusted model (see Supplementary Table S1).

**Table 2:** Association between uterine fibroids and pre-eclampsia.

**Model**	**Covariables in model**	**OR**	**95% CI**	***P-*value**
1	Univariable	2.20	1.20–4.03	0.011
2	Age, BMI, ethnicity	2.01	1.04–3.88	0.037
3	Age, BMI, ethnicity, FPG, dyslipidemia, serum creatinine, age at menarche, primipara	2.22	1.03–4.78	0.041
4	Model 3 + gestational age	2.66	1.18–5.99	0.018
5	Model 4 + BP in early gestation	3.02	1.20–7.60	0.019

Results were showed as odds ratio and 95% confidence interval from logistic regression model. BMI, body mass index; FPG, fasting plasma glucose; BP, blood pressure.

### Sensitivity and subgroup analyses

**Figure 1:**
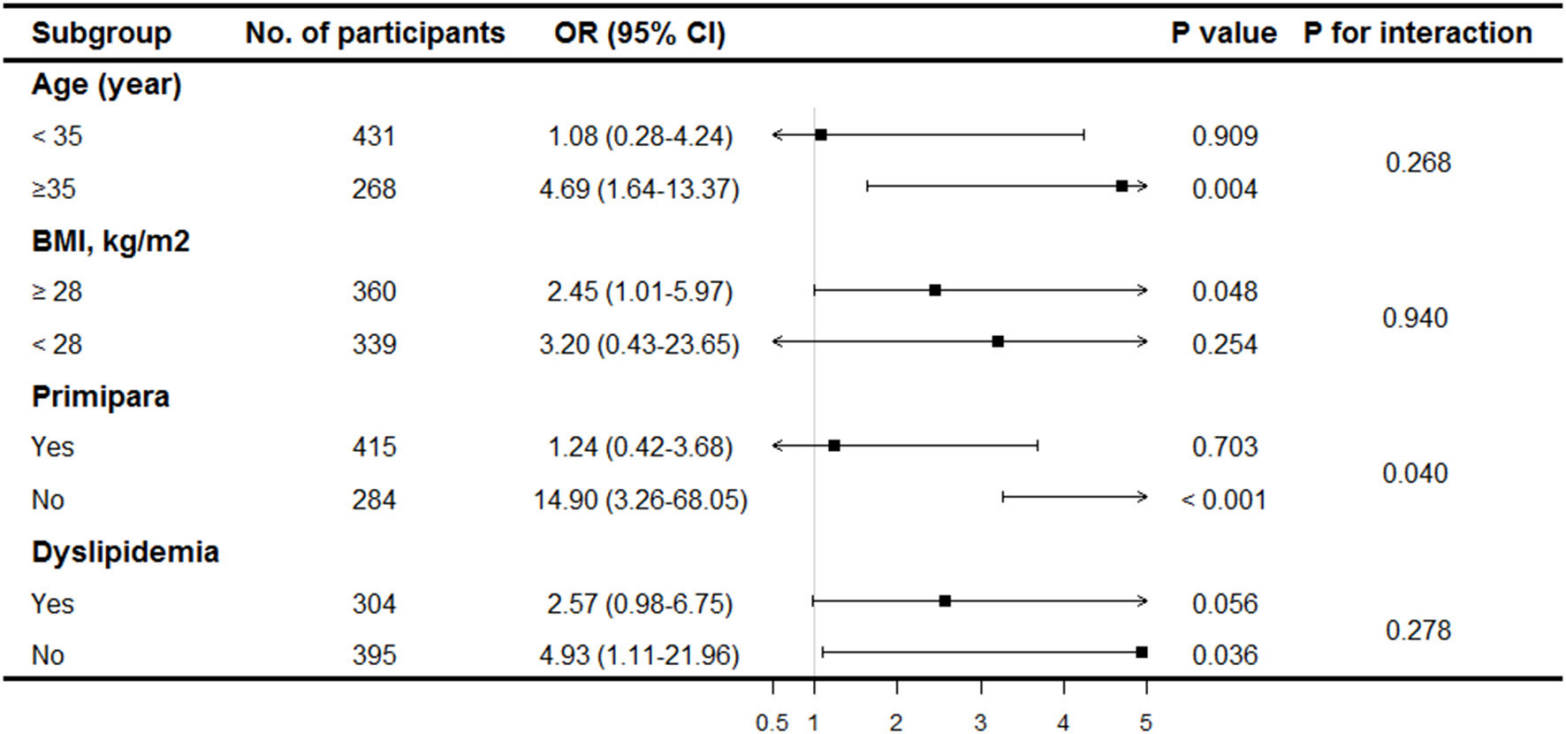
Stratification analysis of association between uterine fibroids and pre-eclampsia. Results were derived from multivariable logistic regression model adjusted for age, BMI, ethnicity, FPG, dyslipidemia, serum creatinine, age at menarche, primipara and gestational age.

By excluding patients with diagnosed gestational diabetes, dyslipidemia and self-reported fibroids, sensitivity analyses confirmed the robustness of the results (Table 3). The odds of PE in women with uterine fibroids were nearly three to five-folds than in those without. Subgroup analyses were performed to further evaluate the homogeneity of the association between uterine fibroids and PE. Similar trends were found in different subgroups of BMI and dyslipidemia (Figure 1). The association was stronger in women aged ≥35 years. In addition, a significant interaction was discovered between uterine fibroids and obstetric history. A stronger association was observed in multiparas, whereas this association disappeared in primiparas.

**Table 3:** Sensitivity analysis for association between uterine fibroids and pre-eclampsia.

**Sensitivity analyses**	**OR**	**95% CI**	***P-*value**
Excluded those with gestational diabetes			
Univariable model	2.54	1.33–4.86	0.005
Full-adjusted model	2.92	1.20–7.08	0.018
Excluded those with dyslipidemia			
Univariable model	3.92	1.21–12.68	0.022
Full-adjusted model	4.93	1.11–21.96	0.036
Excluded those with self-reported fibroids			
Univariable model	2.10	1.07–4.14	0.032
Full-adjusted model	3.29	1.39–7.81	0.007

Results were showed as odds ratio and 95% confidence interval from logistic regression model. Full-adjusted model included age, BMI, ethnicity, FPG, dyslipidemia, serum creatinine, age at menarche, primipara, and gestational age. BMI, body mass index; FPG, fasting plasma glucose.

## Discussion

PE is a major cause of morbidity and mortality in pregnant women and perinatal infants, and its incidence is increasing continuously (19). PE is often detected in the middle and third trimesters and may result in termination of pregnancy, due to treatment limitations (20). It would be beneficial to identify pregnant women who are at high risk of developing PE through screening for risk factors. In this study, we discovered that the presence of uterine fibroids in pregnant women was significantly associated with higher odds of PE. These findings may contribute to the early detection of pregnant women at high risk of PE and implementation of appropriate prevention measures.

Uterine fibroids are the most common benign tumors of the female reproductive system and have been reported to be associated with higher BP levels and hypertension in non-pregnant populations. Takeda et al. have reported in a cross-sectional study that the prevalence of hypertension was significantly higher in patients with uterine fibroids than in those without them (14.6 vs. 0.63%, *P* < 0.001) (11). A case-control study further confirmed the results. After adjusting for confounding factors, such as age, BMI, and race, women scheduled for fibroid surgery had a more than two-fold increased risk of developing hypertension than the control group (13). Fibroid-related inflammatory response and cytokine imbalance may serve as underlying mechanisms for uterine fibroids and hypertension. Patients with uterine fibroids have been demonstrated to be in a condition of chronic inflammation, with increased production of inflammatory factors and activation of the oxidative stress response, leading to impaired endothelial function, which is one of the main mechanisms of hypertension (21, 22). Furthermore, some molecules secreted from fibroids may contribute to the association of uterine fibroids with increased BP levels. Notably, vasodilators, such as endothelin-1, are increased, and vasoconstrictors, such as prostaglandin I-2, are decreased in fibroid tissue and blood circulation (23, 24). However, the association between uterine fibroids and PE has rarely been explored. Chen et al. recently reported in a cohort study that uterine fibroids in early pregnancy were associated with an increased risk of hypertensive disorders of pregnancy (25). Our study, which specifically focused on PE as defined by ISSHP recommendations, reconfirmed this association and extended the generalization of the results. It is noteworthy that similar results were observed (OR = 3.02 in the present study and hazard ratio = 2.95 in the previous study). Taken together, BP monitoring and PE preventive measures may be required for pregnant women with uterine fibroids.

The occurrence and development of PE can be divided into two stages according to the pathophysiological processes. In the early stages of pregnancy, defects in spiral artery remodeling lead to ischemia, hypoxia, and poor placental formation. The degree of spiral artery remodeling is closely related to the occurrence of PE (26). Systematic endothelial activation and vasoconstriction in the middle and third trimesters result in increased BP and other abnormal manifestations. Notably, fibroids undergo rapid and remarkable growth during pregnancy, particularly in the first trimester (17, 27). Therefore, it is reasonable to speculate that the rapid expansion of fibroids in early pregnancy may result in poor placental perfusion by compressing uterine blood vessels, which contributes to the increased risk of PE. Moreover, molecules secreted from fibroids may induce inflammation, oxidative stress response, and endothelial dysfunction and are involved in the development of PE. However, studies with a larger sample size are required to further explore the potential mechanisms.

This study had several limitations that warrant discussion. First, although it could be speculated by case-control design that fibroids are present pre-pregnancy, prospective and interventional studies are required to verify the causal association between uterine fibroids and incident PE. Second, subgroup analyses based on the type, size, and number of fibroids were not performed. The association between fibroid subtypes and PE may differ, and this could provide clues to the mechanisms. Third, other potential confounders, such as pre-pregnancy BP and family history of PE, were not adjusted, which may have biased the results. Fourth, although conducted in a regional tertiary hospital, the generalizability of this study requires further verification in multicenter studies.

In conclusion, uterine fibroids are significantly and independently associated with higher risk of PE in pregnant women. The presence of uterine fibroids in early pregnancy may indicate a high risk of developing PE in the middle and late trimesters. The effect of pre-pregnancy myomectomy on PE prevention is worth further exploring.

## Data availability statement

The data that support the findings of this study are available upon request from the corresponding author. The data were not publicly available because of privacy or ethical restrictions.

## Ethics statement

The studies involving human participants were reviewed and approved by the Ethics Committee of the People's Hospital of Xinjiang Uygur Autonomous Region. The patients/participants provided their written informed consent to participate in this study.

## Author contributions

LG and ML contributed to the study design and statistical analysis. LG, ML, HS, and YH analyzed the data together and drafted the manuscript. LG and HS participated in data collection. All authors have read and approval the final manuscript.

## Conflict of interest

The authors declare that the research was conducted in the absence of any commercial or financial relationships that could be construed as a potential conflict of interest.

## Publisher's note

All claims expressed in this article are solely those of the authors and do not necessarily represent those of their affiliated organizations, or those of the publisher, the editors and the reviewers. Any product that may be evaluated in this article, or claim that may be made by its manufacturer, is not guaranteed or endorsed by the publisher.
