# Supplementary material for: Uterine fibroids are associated with increased risk of pre-eclampsia: A case-control study
Source: Front Cardiovasc Med. 2022 Oct 18;9:1011311. doi: 10.3389/fcvm.2022.1011311 (PMC9623039; doi:10.3389/fcvm.2022.1011311)
Supplement: Supplementary file 1 [file Table_1.DOCX]

SUPPLEMENTARY TABLE

**Uterine fibroids are associated with higher risk of pre-eclampsia: a case-control study**

**Short title:** Uterine fibroids and pre-eclampsia

**All author’s names:** Lina Gong^1*^, Meng Liu^1*^, Haiheng Shi^1^, Ying Huang^1^

**Author’s affiliations:**

^1^ Obstetrics department of People’s Hospital of Xinjiang Uygur Autonomous Region, Urumqi, China.

^*^ These authors contributed equally to this work.

**Correspondence to:** Meng Liu, Obstetrics department of People’s Hospital of Xinjiang Uygur Autonomous Region, 91 Tianchi Road, Urumqi, Xinjiang 830001, China. E-mail: m18684703035@163.com

**Table S1** Collinearity test of included variables

| Variables | Tolerance | Variance inflation factor |
| --- | --- | --- |
| Uterine fibroids | 0.943 | 1.060 |
| Maternal age | 0.793 | 1.261 |
| Body mass index | 0.887 | 1.127 |
| Ethnicity | 0.971 | 1.030 |
| Blood glucose | 0.975 | 1.025 |
| Dyslipidemia | 0.850 | 1.176 |
| Serum creatinine | 0.950 | 1.052 |
| Age at menarche | 0.972 | 1.029 |
| Primipara | 0.835 | 1.198 |
| Gestational age | 0.938 | 1.066 |
| SBP in early gestation | 0.395 | 2.534 |
| DBP in early gestation | 0.388 | 2.575 |
